# Assessment of Stress by Serum Biomarkers in Calves and Their Relationship to Ultimate pH as an Indicator of Meat Quality

**DOI:** 10.3390/ani11082291

**Published:** 2021-08-03

**Authors:** Susana García-Torres, María Cabeza de Vaca, David Tejerina, María Pilar Romero-Fernández, Alberto Ortiz, Daniel Franco, Miguel Angel Sentandreu, Mamen Oliván

**Affiliations:** 1 Meat Quality Area, Centro de Investigaciones Científicas y Tecnológicas de Extremadura (CICYTEX-La Orden), Extremadura Regional Council, 06187 Guadajira, Badajoz, Spain; merycv@hotmail.com (M.C.d.V.); tejerinabarrado@yahoo.es (D.T.); mpromero@unex.es (M.P.R.-F.); alberto.ortiz@juntaex.es (A.O.); 2 Centro Tecnológico de la Carne de Galicia, Av. de Galicia Nº 4, Parque Tecnológico de Galicia, 32900 San Cibrao das Viñas, Ourense, Spain; danielfranco@ceteca.net; 3 Instituto de Agroquímica y Tecnología de Alimentos (CSIC), Avenida Agustín Escardino, 7, 46980 Paterna, Valencia, Spain; copete@iata.csic.es; 4 Servicio Regional de Investigación y Desarrollo Agroalimentario (SERIDA), Ctra AS-267 PK19, 33300 Villaviciosa, Asturias, Spain; mcolivan@serida.org

**Keywords:** farm management, breed, transport, mixing of animal, slaughter time

## Abstract

**Simple Summary:**

The routine handling of cattle during the rearing period and during antemortem events is an inevitable source of animal stress that can have negative impacts on the quality of meat, resulting in economic losses for the meat industry. This study assesses the breed, the farm management system, and the pre-mortem handling of animals and their impact on serum stress biomarkers, as well as their relationship with pH_u_. The findings indicate that breed, together with farm management, had the highest impact; cortisol, lactate, and serum amyloid A were identified as potential stress biomarkers in relation to pH_u_. We believe that these findings might help in the assessment of stress in cattle at the stage prior to slaughter.

**Abstract:**

Seventy-eight calves from Asturiana de los Valles, Retinta, and Rubia Gallega breeds, under extensive and intensive farm systems and animal mixing and non-mixing conditions, and during the transport and lairage in slaughterhouses, were studied. This research aimed to study the effect of breed, farm system and mixing conditions on serum biomarkers (cortisol, lactate, glucose, serum amyloid A, haptoglobin, and C-reactive protein) and their relationship with pH_u_ at slaughter time, and to evaluate the response of the serum biomarkers of calves throughout fattening period. Moreover, this study aims to evaluate the response of the biomarkers in each breed during the fattening period. At slaughter time, cortisol and lactate were affected by BreedxFarm; Retinta showed the opposite pattern to the others and revealed the highest glucose in extensive farm systems. Rubia Gallega in mixing revealed the highest Amyloid A and haptoglobin. Extensive calves in mixing conditions showed the highest glucose. There was a relationship among the variables cortisol, lactate, Amyloid A, and pH_u_. Slaughter time was a major stressor, and the stress response was mainly affected by breed. At slaughter, several biomarkers should be considered.

## 1. Introduction

In animal husbandry, stressful events arise from situations associated with the routine handling of animals and animal–human interactions. These events trigger reactions in animals that translate into physical, physiological, and behavioral stress [1,2], which may have an effect on the ultimate meat quality, leading to the appearance of DFD meat (Dark, Firm, Dry), which is usually related to a high pH at 24 h post-slaughter [3,4].

Animal response to stressful stimuli depends largely on intrinsic factors such as gender [5] and breed, which are important variation factors in the response to stress [6,7,8], as well as extrinsic factors relating to the usual methods of handling in farms and pre-slaughter management [4], such as loading, transportation, and lairage in slaughterhouses [9,10,11]. In addition to this, the production system (whether extensive or intensive) and environmental conditions are extrinsic factors that are responsible for stress [8,12,13], together with the social mixing or regrouping of animals in the farm for transport to the slaughterhouse [14,15,16,17]. However, the psychological and physiological status of the animals or the relationship among animals in the herd’s social behavior can also be a cause of stress [7,12,18,19].

An example would be weaning calves. This is a usual husbandry practice that involves multiple stress factors at psychological, physiological, and nutritional levels [20,21]. These practices may results in an alteration in calves’ immunity, an increase neutrophil counts and in Acute Phase Proteins (APP), such as cortisol, haptoglobin, and serum amyloid A [15,21].

From a physiological point of view, stress causes homeostasis imbalance, which in turn generates a physiological response that is able to trigger the HPA axis (hypothalamic-pituitary-adrenal) in order to reestablish homeostasis. Multiple organs and neuroendocrine hormones are involved [22] in this process. The role of these hormones and blood proteins is to regulate, among others, the behavioral, metabolic, cardiovascular, immune, and gastrointestinal functions [23,24]. Among them, cortisol is considered to be the main stress biomarker, despite its variability and short life span [25,26,27]. Haptoglobin (Hp) and serum amyloid A (SAA) are considered to be very important stress biomarkers in ruminants [28]. 

Asturiana de los Valles (AV), Retinta (RE), and Rubia Gallega (RG) are native Spanish cattle breeds with meat aptitude that have a recognized Protected Geographical Indication (PGI) quality brand known as “Ternera Asturiana” [29], PGI “Ternera Gallega” [30] and “Carne de Retinto” [31]. These account for approximately 75% of the autochthonous quality beef on the Spanish market [32].

These three breeds have adapted to extensive management under grazing conditions. The AV and RG breeds are raised in the North of Spain (Asturias and Galicia, respectively) and are easy to handle, while the RE breed is the most important breed in the Southwest of Spain (Extremadura) and is difficult to handle due to its wild temperament; this is even a trait that is included in the national selection program of the Retinta breed [33]. The RG breed is one of the most important local cattle breeds in Spain [34] and has a good meat yield (>80%); unlike other cattle breeds, it produces light meat that is well valued on the market [35]. The AV breed is a late-maturing breed with a high growth rate, high muscle percentage, and low-fat content [36,37,38,39]. The RE breed is rustic and has adapted to extensive production systems in the dehesa ecosystem (free-range).

Stress biomarkers can hypothetically react differently to pre-slaughter stress depending on the breed, farm management system, and the conditions that animals are kept in during transportation, and they may be useful to identify DFD meat at the preslaughter stage. The aim of this paper is to evaluate the effects of breed (AV, RE, and RG), farm management system (Extensive “E” vs. Intensive “I”), and pre-slaughter handling (animal mixing during transport and lairage prior to slaughter: Mixing “M” vs. Non-Mixing “NM”) on the main serum biomarkers at slaughter time in calves in order to study their relationship with ultimate pH as an indicator of meat quality. Additionally, the aim is to evaluate the response of the serum biomarkers of calves according to breed from the start of their fattening period until slaughter.

## 2. Materials and Methods

### 2.1. Animals and Experimental Management

Seventy-eight calves (24 AV, 22 RE, and 32 RG) were used in this study. AV calves were reared in the Finca “La Mata-Grado”- belonging to SERIDA in Asturias, RE-calves in the Finca “Valdesequera”– belonging to CICYTEX in Extremadura and RG calves COREN cooperative in Ourense, with the consent of the breeders for the study. In each breed, calves were managed with their mothers from birth to weaning time, and at 100 days before slaughter, they were assigned to two different farm management systems: (1) Intensive “I” indoor management (8 kg/day of concentrate (84% barley meal, 10% soya meal, 3% fat, 3% minerals, vitamins, and trace elements) + 2 kg/day of barley straw) or (2) Extensive “E” outdoor management (grazing on natural pasture + 3.5 kg/day of supplementation with a concentrate as described above). Animals of the Intensive management were managed indoors, in two pens of 6 × 6 m (6 m^2^ per animal), while animals of the Extensive group were managed outdoors in two 1.5 ha plots (6 animals per plot; 2500 m^2^ per animal). The experimental procedures to which the animals were subjected during the experiment period were in compliance with the RD 53/2013, which establishes that no authorization is required for practices carried out for recognized zootechnical purposes (Art 2.5d) and those that do not cause more pain than the introduction of a needle (Art 2.5f).

All animals were slaughtered at the age established by the local market for Protected Geographical Indication (PGI) beef for each breed, i.e., 9 months for RG-breed animals and 15 months for the AV and RE breeds.

When the animals reached slaughter conditions and left the farm for the slaughterhouse, a pre-slaughter stress factor was applied to half of the calves from each breed (AV, RE, and RG) and management system (E and I): half of the animals remained together all the time, during the fattening period, transport and lairage time, named “non-mixing treatment (NM)”, whereas the other half were mixed with unfamiliar individuals from other farmers during transport and lairage stages, named “mixing (M)”. The trip duration from farm to slaughterhouse was of the maximum duration of 75 min and was in accordance with Council Regulation (EU) Nr. 1/2005, which relates to protecting the welfare of animals during transportation. In the commercial slaughterhouses, calves were stunned with a captive bolt and slaughtered by immediate exsanguination according to current EU regulations (Council Regulation (EC) No 1099/2009).

### 2.2. Blood Collection

Blood samples during the fattening period were taken from each animal in the morning by coccygeal venipuncture into 10 mL evacuated tubes (Vacutainer, BD), without anticoagulant at three different times of cattle life (T0: weaning and starting of the fattening period; T50: half-way through the fattening period, and T100: final fattening period). Finally, blood samples were collected in two tubes of 10 mL without an anticoagulant during exsanguination (Ts) in the slaughterhouse. In the laboratory, serum samples were obtained by blood centrifugation at 3000× *g* for 15 min and kept in aliquots at −80 °C until subsequent analysis of the biomarkers.

### 2.3. Blood Biochemical and Physiological Parameters

The serum parameter levels were determined using various commercial kits. All the samples were assayed in duplicate and averaged out. In particular, the biomarkers under analysis were the following:

- Stress hormone: cortisol (C) was assayed by the competitive Enzyme-Linked Immunosorbent Assay (ELISA), i.e., the kit ADI-900-071 from Enzo Life Sciences (Lausen, Switzerland). The measurements were made using a colorimetric method, which detects the final concentration of cortisol conjugated with alkaline phosphatase at 405 nm. Absorbance was measured in a UV/Vis spectrophotometer for microplates (Multiskan GO, Thermo Scientific™, Vantaa, Finland).
- Biomarkers of the energetic metabolism: Both the lactate (L) and glucose (G) parameters were analyzed using enzymatic-ultraviolet determinations kits, based on the quantifications of final NADH or NADPH produced after enzymatic digestions (R-Biopharm, Darmstadt, Germany). The readings were taken at 340 nm absorbance with a Cary 60 UV-Vis Spectrophotometer (Agilent Technologies ™, Santa Clara, United states).
- Biomarkers of the inflammatory process: serum amyloid A (SAA), haptoglobin (HP), and C-reactive protein (CRP). SAA was determined by a colorimetric sandwich ELISA kit (Phase serum amyloid A, Tridelta Ltd., Maynooth, County Kildare, Ireland). The spectrophotometric measurements were made in a UV/Vis spectrophotometer for microplates (Multiskan GO, Thermo Scientific™) at 450 nm absorbance while the assessment of Hp was carried out using a colorimetric enzymatic kit assay (Phase haptoglobin Assay, Tridelta Ltd., Maynooth, County Kildare, Ireland). The measurements were made in a spectrophotometer at 630 nm absorbance (Multiskan GO, Thermo Scientific™). The assessment of the CRP was carried out by a kit test in order to discriminate serum CRP-positive samples (CRP ≥ 6 mg/L) (Biosystems C-Reactive protein), based on the agglutination of latex particles coated with a C-reactive anti-protein.

### 2.4. Meat Ultimate pH (pH_u_)

pH was measured on the *Longissimus thoracis et lumborum* (LTL), taken from the left half-carcass at the level of the 6th rib at 24 h post-mortem using a penetration electrode, coupled with a temperature probe (Crison pH-meter mod. MicropH 2001). 

### 2.5. Statistical Analysis

The raw data were scrutinized for data entry errors and outliers. The effect of breed “B” (AV, RE, and RG), farm management systems “F” (I vs. E) and pre-slaughter handling during transport and lairage “PSH” (M vs. NM), and their interactions on serum biomarkers (SAA, C, G, L, Hp, and CRP), were analyzed by a multivariate analysis of variance using the General Linear Model (GLM) procedure of SPSS (v 15.0 2006, SPSS Inc., Chicago, IL, USA). This considered each animal as the experimental unit and included the animal’s age as a covariate in the model. Additionally, a one-way ANOVA test was used to study the effect of blood collection times “T” (T0, T50, T100, and Ts) on each breed individually. The significance level for differences among treatments was set at 5% (*p* = 0.05) according to Tukey’s HSD test, and the significant differences in the post hoc test were used to compare the various groups (AV, RE, and RG for Breed and T0, T50, T100 and Ts for blood collection times). 

The relationship between the variables evaluated in serum biomarkers at Ts and the meat pH_u_ parameters were calculated using Pearson’s correlation coefficient (r) with a significance level of *p* = 0.05. A principal component analysis (PCA) was performed in order to study the relationships among the serum biomarkers at Ts and pH_u_ under study.

## 3. Results and Discussion

### 3.1. Biomarkers at Slaughtering Time (Ts)

The effect of breed (B), the farm management system (F), and the pre-slaughter handling (PSH) on the blood biomarkers of stress at slaughter time are shown in Table 1. Most of the analyzed biomarkers were affected by the interaction of breed with the other analyzed factors: Breed and farm management (B×F) and Breed and Pre-slaughter handling (B×PSH).

#### 3.1.1. Stress Hormone

**Figure 1:**
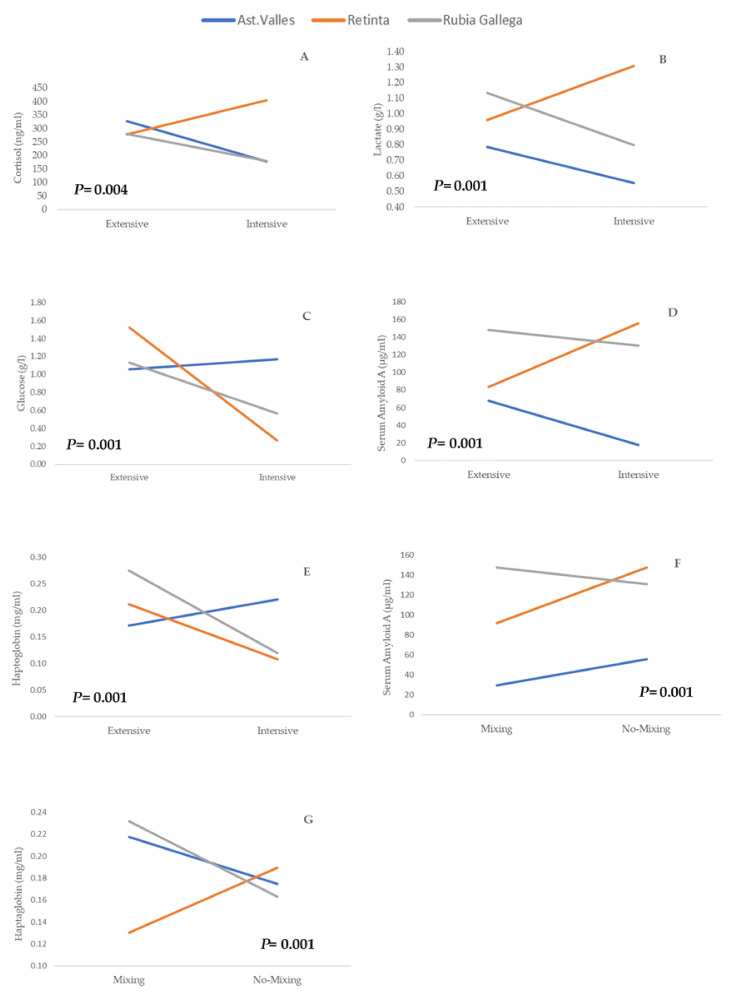
Interaction effects on serum biomarkers at slaughtering time (Ts). Interaction breed x farm management system on (**A**) cortisol, (**B**) lactate, (**C**) glucose, (**D**) serum amyloid A, and (**E**) haptoglobin. Interaction breed x pre-slaughter handling on (**F**) serum amyloid A and (**G**) haptoglobin. Extensive (*n* = 39); intensive (*n* = 39); mixing (*n* = 40); no-Mixing (*n* = 38).

The cortisol levels at slaughter time were higher than those reported in other studies with cattle [40,41,42]. The interaction between breed and farm management system on cortisol values was significant (*p* = 0.004), with AV and RG calves showing the same behavioral pattern, with lower cortisol levels in the blood of animals from the Intensive system, while the opposite behavior in RE calves was observed (Figure 1A). However, cortisol did not show significant differences due to the interaction between pre-slaughter handling with the breed (*p* = 0.364) and with the farm management system (*p* = 0.295) interactions, which is a fact that allows us to analyze these effects independently. In this case, cortisol was only affected by breed, and RE calves showed higher cortisol values than AV and RG calves (Table 1). It is difficult to establish a reference value for cortisol in response to pre-slaughter stress because there are many factors to be taken into account. The differences identified among breeds highlight the link between activation of the HPA axis by stressors and genetics [43,44], which includes temperament [45,46,47]. RE calves showed the highest cortisol value at slaughter time, and even though we have not measured “temperament” as a feature in this research, unlike the aforementioned authors [47] have, it is well-known that the RE breed has a wilder “temperament” than AV and RG breeds, a factor that could explain this finding. However, the AV and RG breeds are improved breeds that provide easier handling and are genetically closer [48], and show the lowest cortisol levels at slaughter time. 

#### 3.1.2. Energetic Metabolism Biomarkers

Concerning lactate (L), the interaction between breed and farm management systems was significant (*p* = 0.001). The response of the lactate (L) biomarker in the two farm management systems was similar for AV and RG calves, with higher lactate values in the extensive rather than the intensive system (Figure 1B). However, the RE calves displayed the opposite behavior, showing the highest lactate concentrations in the intensive system. The variations identified in the results could be attributed to the fact that the animal responses to stressful situations are governed by a complex interaction of genetic factors, including temperament and previous experiences, such as farm management [49]. Serum lactate is deemed to be an acute stress biomarker, and its reference values are in the range of 0.6- 2.2 mmol/L (0.054–0.198 g/L) [2]. Thus, the resulting levels were above the reference values (Table 1), indicating an acute stress response that, mediated by the production of catecholamines, led to increased blood lactate concentrations in AV and RG calves reared in extensive systems and in RE calves reared in intensive conditions. The increase in lactate level in the RE breed has been previously described in reactive animals by some authors, who found a higher level of plasmatic and muscular lactate, meaning that these animals mobilize more muscle glycogen. The RG calves under the extensive management system at slaughter time showed the highest level of lactate, and in this case, this cannot be justified by temperament [50] but could be a response to the physical stress of these animals, which is supported by the SAA concentration (Table 1). This result is in line with the work by Alsemgeest et al. [51], who indicated the sensitivity of this biomarker to the lack of physical welfare in calves.

Regarding glucose levels at slaughter time, the interaction of breed and farm management system on glucose at slaughter time was significant (*p* = 0.001), indicating that the glucose levels, as a stress response to the farm management system, must be assessed by breed (Figure 1C). The glucose concentration values for the RE and RG breeds were higher in E than in the I management systems, while in the case of AV calves, no differences were found on account of the farm management system. The findings showed that the glucose levels of RE-E calves are the highest, and therefore, these were the most sensitive animals to handle and their relationship with humans during managements. The glucose reference level after transport to the slaughterhouse and during slaughter, reported by Tadich et al. [11], was 6 mmol/L (1.080 g/L). The results of serum glucose found in the RE calves, regardless of the farm management, and for RG-E calves, were clearly above the values reported by these authors. Plasma glucose levels are directly related to the nutritional status of an animal, but they can also increase as a response to stress, as catecholamines are released by the adrenal glands, and liver glycogenesis is stimulated, which increases the availability of plasma glucose [52]. Our findings show an increase in glucose according to the cortisol increase that was also observed (Figure 1A). These results indicate that changes in the glucose concentration were caused by stress due to the handling of the animals throughout the pre-slaughter process [11], and the breed and farm management system used with the animals must be taken into account. The AV calves did not show differences by farm management effect, but the glucose levels in the RE and RG calves decreased when the animals were under I management, indicating a greater response (higher levels of glucose) to sensitivity at the slaughter time of calves from extensive systems in these breeds.

The interaction between breed and pre-slaughter was not significant on cortisol, lactate, and glucose (*p* = 0.364, *p* = 0.925, and *p* = 0.148, respectively), making it appropriate to analyze these effects independently. Thus, the pre-slaughter handling had an effect on glucose, being higher in mixing than non-mixing animals, whereas the breed was the main effect on cortisol and lactate. In this regard, the RG breed showed a low response to stress in accordance with the cortisol value, but they were more sensitive to the exercise activities developed during pre-slaughter management, as can be seen at the lactate level (Table 1). However, the glucose level was the differentiating biomarker of the stress due to pre-slaughter handling under the study (mixing or non-mixing).

##### Inflammatory Process Biomarkers

Regarding the determinations of SAA, the interactions between breed and farm management and pre-slaughter handling (B×F and B×PSH) were significant (*p* = 0.001), and consequently, the interpretation of the main effects was more complex. These interactions are depicted in Figure 1D,F, respectively. Both the AV and RG breeds showed different behavior with respect to the RE breed. It is remarkable to note that the SAA concentration found in AV calves at slaughter time was the lowest in all cases. Our findings demonstrated that the effects of farm management and pre-slaughter handling should be assessed separately within each breed. The RE calves at slaughter time showed high levels of SAA in calves from intensive management systems and from NM treatment during pre-slaughter handling. According to Miranda-de la Lama, Villarroel, and María [53], high values of SAA indicate sensitivity to the lack of physical welfare in calves, such as pre-slaughter conditions. Our results were higher than those reported by Lomborg, Nielsen, Heegaard, and Jacobsen [54], who measured it in calves under physical stress. This biomarker could be useful in assessing animal welfare in various production systems, indicating a lack of physical welfare in calves due to stress conditions such as during pre-slaughter management.

Concerning the haptoglobin biomarker, it also proved to be a highly complex situation from interaction analysis of the breed with farm management (B×F; *p* = 0.001) and with pre-slaughter handling (B×PSH; *p* = 0.006). Figure 1E of breed and farm management interaction showed that the haptoglobin values in RE and RG calves under extensive systems were higher than those in intensive systems, while in the AV breed, the response was the opposite. Regarding the interaction between breed and pre-slaughter handling (Figure 1G), the RE breed showed a different response due to pre-slaughter handling because the RE calves had lower haptoglobin levels in mixing than in non-mixing conditions. According to the literature, our results were above 0.1 mg/mL, which is deemed to be the reference value [55]. As far as we know, the literature is scarce in reporting the relationship between the farm management system and the response to stress by studying serum biomarkers in calves at slaughter. The increase in haptoglobin is related to situations of poor welfare status [15]. This biomarker is synthesized by hepatocytes in response to macrophage cytokines in the case of tissue damage, inflammatory processes, infection, and in response to stress [56]. 

SAA and haptoglobin are plasmatic proteins whose concentration can be altered, subject to stress conditions, and in relation to an immune response to the cytokines produced by macrophages. Therefore, these proteins are useful as biomarkers for the inflammatory processes, such as disease, in both human and animal health [51]. In ruminants, SAA and haptoglobin are considered to be the most important Acute Phase Proteins [28,57]. However, there is controversy in the literature about their roles. Our results agree with Joshi et al. [58], who considered haptoglobin to be more sensitive than SAA because it raises its levels more rapidly than SAA, which requires a stronger stressor to be applied for a longer time for its serum to increase in the bovine respiratory disease of dairy calves. In contrast, other authors [15,51,59] suggest that haptoglobin is less sensitive and reacts more slowly than SAA.

**Figure 2:**
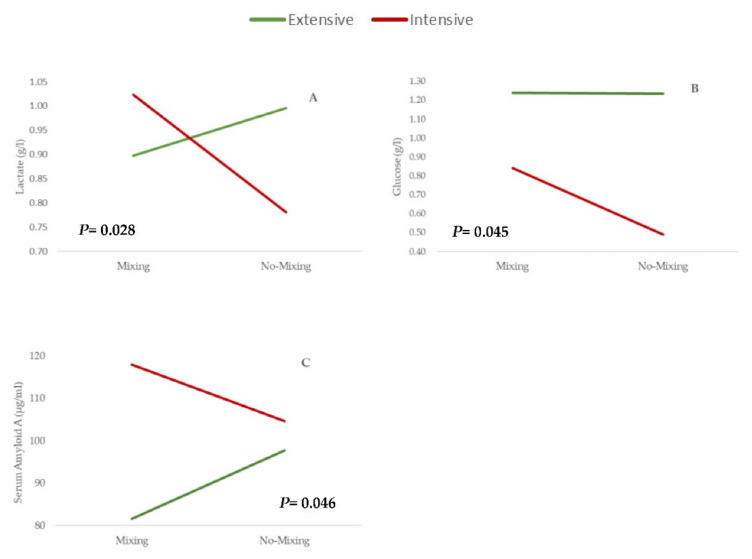
Interaction farm management system x pre-slaughter handling at slaughtering time (Ts) on (**A**) lactate, (**B**) glucose and (**C**) serum amyloid A. Mixing (*n* = 40); no-Mixing (*n* = 38).

The response in view of the pre-slaughter handling events (transport and lairage in the slaughterhouse) was impacted by the farm management system (E or I) and the animal’s previous experience (i.e., frequency of human contact, and consequently, the specific psychology and physiological status of the animals). Pre-slaughter handling during the fattening period affects the response of animals. Some biomarkers, such as lactate, glucose, and SAA, were affected by the interaction between the farm management and pre-slaughter handling (Figure 2). The lactate and SAA levels in response to the pre-slaughter events depended on the origin of calves in the extensive and intensive systems (Figure 2A; *p* = 0.028 and Figure 2C; *p* = 0.046, respectively). The stress factors arising during pre-slaughter handling, when animals were mixed, showed that the intensive calves suffered from increased plasma lactate and SAA. These biomarkers, lactate and SAA, are related to stress from exercising, and are a response for the opposed behavior, depending on the farm management system effect, it is not clear. According to Coombes, Gardner, Pethick, and McGilchrist [60], the origin of the calves also impacts the response of the stress biomarkers to pre-slaughter handling, in addition to the differences among individuals in stress-related behavioral and physiological status [61]. The I-M calves showed a higher response at slaughter time than I-NM calves. Initially, these animals were at the production phase, in conditions adapted to their handling, human presence, and exercise, which allowed them to be confined, so mixing with non-family animals and the situation of transport and lairage to the slaughterhouse could explain the increase in the levels of lactate and SAA. This result could also be expected from calves under the E management system, but this was not the case. These calves’ behavior was different because they had the chance to do more exercise for longer periods of time, the chance to relate to humans to a lesser extent, and they had become accustomed to facing other types of factors in nature, such as the weather or the presence of other wild animals. Overall, they had more adverse behavioral reactions to handling and chute restraints. In that respect, Gruber et al. [62] reported a higher presence of plasma lactate at slaughter in these types of animals. In order to understand these findings, it would be necessary to carry out further studies to include other stress indicators in each of the pre-slaughter handling phases, such as calves’ vocalization, as reported by Probst et al. [63].

Concerning the glucose levels, the interaction between farm management and pre-slaughter handling was significant (Figure 2B; *p* = 0.045), and the stressors during the transport and lairage in the slaughterhouse did not affect the extensive calves, but in all cases, they showed the highest glucose values. The extensive system involves less handling and human approaches. The handling of animals in pre-slaughter, transport, and the time in the slaughterhouse are highly stressful situations that lead to an increase in glucose, whether the animals are mixed or non-mixed. However, the pre-slaughter condition affected the glucose level, which rose in mixing calves under intensive management. Stress-related physiological changes, such as mixing animals during transport and lairage, are associated with changes in blood glucose levels [40]. 

The interaction between farm management and pre-slaughter handling effect on haptoglobin values did not show significant levels (*p* = 0.076); therefore, the main effects can be examined independently. The type of farm management significantly affected (*p* = 0.001) the haptoglobin values, with the highest value being identified in E-calves, while there was no significant influence of pre-slaughter handling (M/NM; *p* = 0.325) on this parameter. This finding indicated that E calves were more sensitive to pre-slaughter handling than I calves. Therefore, the findings reporting that there was no significant difference among the cortisol concentrations of the calves, regardless of the farm management system used, probably indicates that the pre-slaughter handling process stressors were important for all the animals. This result was also obtained by Averós et al. [40], in calves reared under an extensive system and after commercial transport. They found that, after long transportation periods of the animals, the haptoglobin levels reflected a phase of acute stress, although the cortisol values did not experience a significant change. However, the SAA levels were seen to be higher, regardless of the F system, and only the Hp concentration was higher in extensive calves than intensive calves. This fact could suggest differences in the response kinetics of both SAA and haptoglobin, as reported by Lomborg et al. [54]. In order to clarify this issue, it would be necessary to carry out research on the hepatocytes’ physiology and the production of the Acute Phase Proteins against stress situations.

CRP has been described by Schrödl et al. [64] as an inflammatory biomarker, especially in humans, and indicated that this is not a valid marker for cattle, which is in line with the results of the present study of slaughter time. As can be seen in Table 1, the three factors herein analyzed did not significantly affect the CRP test (% positive), as it can be seen in the lack of significant differences in our results (Table 1).

Livestock transportation is a necessary situation in the meat industry chain. This process, under commercial conditions, begins with the handling of the animals in order to load them into the truck, which will carry them to the slaughterhouse, and continues with handling in the slaughterhouse, the lairage time and the handling of the animals up to the stunning box, and finally slaughtering. The effects of different transport conditions on the stress of animals have been widely understudied [11,12,15,42,65]. In the present study, we can consider that the duration of transport from the farm to the slaughterhouse in all cases was “short” according to the criteria set out by Averós et al. [40], since the maximum duration was 75 min. This stressful situation can be aggravated by the common practice of mixing unknown calves together during transportation, thereby introducing social disruption that accentuates the degree of stress among them [12,53], which in turn has a great impact on the final meat quality [8,66,67,68]. 

The production system (i.e., the farm management system) was revealed to be of great importance at the slaughter time on the calves, depending on the breed analyzed. Thus, some stress biomarkers showed different responses depending on the breed that faces transport and lairage in the slaughterhouse, as well as the moment of slaughter itself. The highest values for cortisol, lactate, and SAA were seen in RE calves at slaughter time from intensive management systems, while the AV and RG calves showed the highest values under extensive management systems. Concerning glucose and haptoglobin levels, the response was also different depending on the breed. The results obtained seem to indicate that the link between breed and the traditional production system is largely important in the stress management process, from farm to slaughterhouse. The different temperaments of the breeds under study should be noted, as was indicated above for the RE breed. In contrast, the AV and RG breeds are genetically closer [48], and their temperament is calmer, non-aggressive or reactive, particularly the RG breed [50]. Other authors [8,47] indicated the importance of breed on stress response, but to the best of our knowledge, there is a lack of information concerning the effect of the interaction between the farm management system and the breed on the pre-slaughter stress biomarkers.

The increase in catecholamines and cortisol in stress situations causes the depletion of glycogen reserves due to the need to increase the availability of glucose as a source of energy. The result is a decrease in the production of lactic acid in the post-mortem muscle, giving rise to higher ultimate pH values ≥ 6.0, which in turn results in an abnormal conversion of muscle into the meat that is known as DFD. In the current study (Table 1), although some animals showed a pH_u_ ≥ 6.0, the average values of pH_u_ obtained were within the values considered to be normal (pH_u_ < 6.0). 

The breed effect showed a significant difference in pH_u_ (*p* ≤ 0.001). The RE breed showed higher average pH_u_ values compared to AV and RG (Table 1). According to the classification proposed by Ijaz, Li, Zhang, Hussain, and Ren et al. [69], the RE calves could be classified as atypical DFD because the average pH_u_ value was 5.73 (5.70 < pH_u_ < 6.09). While this value was the highest of all the values being measured, it was within the normal pH range for beef [70]. Gruber et al. [62] and Coombes et al. [60] indicated that, although cattle of all temperaments have an equal risk of a dark cut, the calves with more reactive temperaments mobilize more glycogen at the time of slaughter than calves with calmer temperaments. This finding would explain the increased pH_u_ in RE calves without the temperament being a reason to obtain DFD meat, although the RE breed can be considered to be the most reactive breed.

Pre-slaughter handling (PSH) not only affects the biochemical parameters of the blood but may also have an impact on the meat quality. Due to the stress situation throughout the pre-slaughter handling, sometimes the antemortem muscle is depleted of its glycogen storage and causes less post-mortem lactic acid accumulation, and a high ultimate pH, as well as a loss in meat quality. This is a serious problem for the meat industry, which gives rise to major economic losses. 

The correlation coefficients were estimated in order to determine the relationship between all the blood biomarkers under study and the pH_u_ at slaughter time (Table 2). Some precautions should be taken in the interpretation of these correlations since the values obtained a Pearson’s correlation coefficient of R^2^ ≤ 0.5 and the linear relationship between the analyzed variables was therefore low. In the present study, the lactate concentration was positively correlated with SAA (r = 0.342; *p* ≤ 0.01) and cortisol (r = 0.430; *p* ≤ 0.001), as well as glucose with haptoglobin (r = −0.383; *p* ≤ 0.001). Lu et al. [3] reported a positive relationship between lactate and cortisol and glucose, although, in the current study, no correlation was identified between lactate and glucose. The positive relationship between the concentration of lactate and SAA could be explained by the fact that both biomarkers are related to physical exercise since the level of lactate in the blood rises rapidly due to physical and behavioral reactions to stress [3], whereas SAA is considered to be a sensitive biomarker for assessing physical well-being [51]. However, the pH_u_ is correlated with different serum biomarkers, such as SAA (r = 0.468; *p* ≤ 0.001), cortisol (r = 0.400; *p* ≤ 0.001) and lactate (r = 0.492; *p* ≤ 0.001). According to Lu et al. [3], a positive relationship between pH_u_, cortisol, and lactate was revealed, although our findings also revealed a positive relation between SAA and pH_u_, which the previous authors did not find. In any case, as aforementioned, although there is a relationship between these parameters, it can be risky to relate the biomarkers that have a low Pearson’s correlation index.

##### Multivariate Analysis

**Figure 3:**
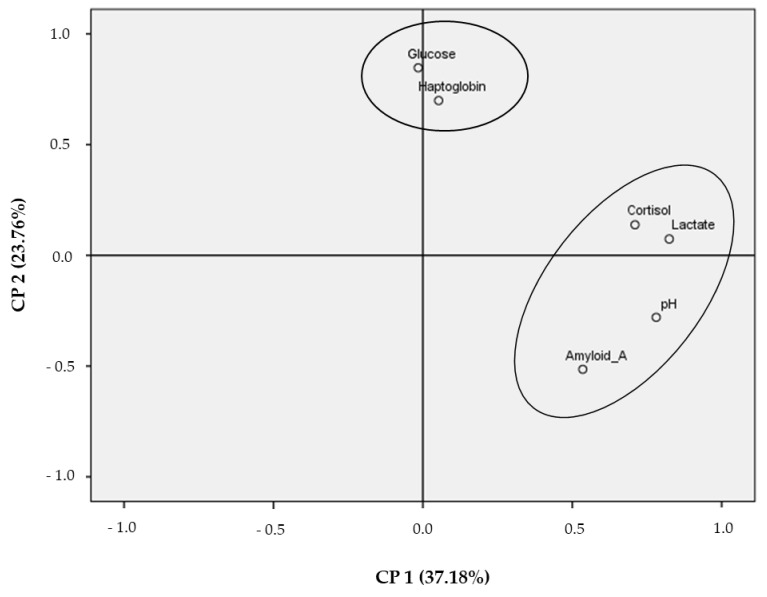
Loading plots for the first two principal components of serum biomarkers at slaughtering time and the pH_u_ of meat, using cortisol, lactate, pH_u_, and serum amyloid A (PCA1) and serum amyloid A (−), glucose, and haptoglobin (PCA2).

In order to study the relationship between the serum biomarkers and pH_u_ as a marker of meat quality, and in order to obtain an overall idea, a principal component analysis (PCA) was carried out. As shown in Figure 3, a multidimensional space, based on these data, is reported in a bi-plot. The total variance explained for the cortisol, lactate, glucose, SAA, haptoglobin, and pH_u_ parameters was 60.94%. The first main component (PC1), which explained the higher percentage of variance (37.18%), was mainly associated with cortisol, lactate, SAA, and pH_u_.

### 3.2. Effect of Blood Collection Times on Serum Biomarkers by Breed

**Figure 4:**
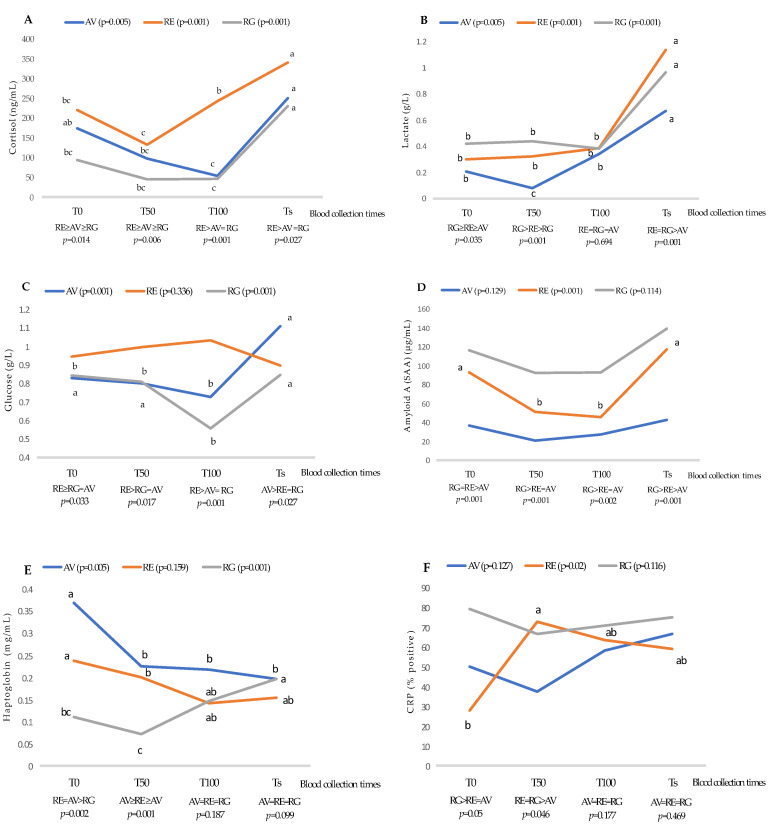
Effect blood collection times by breeds on (**A**) cortisol, (**B**) lactate, (**C**) glucose, (**D**) serum amyloid A, (**E**) haptoglobin, and (**F**) CRP (C reactive protein). AV: Asturiana de los Valles breed (*n* = 24); RE: Retinta breed (*n* = 22); RG: Rubia Gallega breed (*n* = 32); T0: Weaning and starting of the fattening period; T50: Half time of the fattening period; T100: Final fattening period; Ts: Blood samples were collected during exsanguination. Within each breed, different letters mean significant differences between the studied blood collection times for *p* = 0.05 according to Tukey’s test.

The effects of blood collection times (T) on serum biomarkers by breed are plotted in different figures (Figure 4). Regarding cortisol (Figure 4A), higher values were identified at T0 than at T50 and T100, although the highest cortisol value was identified at slaughter time. However, cortisol values for RE calves were higher in all the sampling points, while AV and RG calves showed a similar behavior, mainly when the calves were at the final fattening period. These findings reflect the point made previously, i.e., the difference in temperament among the animals subject to breed. Indeed, RE calves showed higher cortisol values than the AV and RG breeds in all the blood collection times. Furthermore, the cortisol levels at T0 could be related to weaning and the beginning of the fattening period because the weaning phase involves high levels of stress associated with high levels of cortisol, as previously reported [71]. However, for all the calves in the present study, the pre-slaughter handling (transport and lairage in a slaughterhouse) was the most stressful period.

Concerning serum lactate (Figure 4B), there were no variations among the analyzed blood collection times effect in each breed, except at slaughter time. At that time, the lactate levels were the highest, probably as a response to pre-slaughter stress, and specifically due to the exercise undertaken by the animals while being loaded and unloaded on to and out of the truck, in maintaining balance with the movements of the truck during transport, and in the handling itself in the slaughterhouse in relation to the driving of the animals to the stunning box [2,72]. However, the animals that were the most sensitive to exercise stress were RG, but these differences disappeared at T100 among breeds when the animals reached the end of the fattening period, although the levels at slaughter time were the highest in all the breeds. The AV calves showed the lowest lactate level at all the blood collection times, which indicates that this breed has greater adaptability to farm management events than the other two breeds under study.

Regarding the glucose level (Figure 4C), while the RE calves were not affected by blood collection times, in the case of the AV calves, the glucose level rose at slaughter time, and the RG calves showed a decrease in the glucose levels at T100, after the fattening period. The highest glucose levels throughout the production phase were identified in RE calves at slaughter time; meanwhile, serum glucose was higher in AV calves in response to pre-slaughter handling. According to Shaw et al. [52], the increased glucose in animals is related to a rise in cortisol, which leads to an increase in the production of liver glycogenesis in response to stressful situations. In this sense, RE calves showed the highest glucose levels, which were coincidental with the highest cortisol values throughout the production phases. In this breed, the glucose was reduced at slaughter time, while the cortisol was higher. This finding was described by other authors [40,73]. Far from stating that the decrease in the glucose level responds to the fact that the animals were not stressed, we can suggest an explanation based on a genetic factor, as supported by Poleti et al. [44], who reported an association between the polymorphisms of certain genes with changes in the HPA axis activity and the metabolism. 

The level of SAA was only affected by blood collection times in the RE calves (Figure 4D), showing the highest values at T0 and slaughter time. As mentioned above, the RG calves were the most sensitive animals to the lack of physical welfare [51,54]. The only breed showing stress changes due to a physical welfare response was RE, which was more susceptible than AV and RG animals. During the fattening period, the highest SAA levels were at T0, related to the weaning process and the start of the fattening period. After that, this parameter decreased, and it was similar at T50 and T100. The calves at slaughter time again showed higher levels of SAA as a response to the physical stress when faced with pre-slaughter events.

The haptoglobin levels (Figure 4E) were affected by blood collection times in the three breeds under study. Thus, among the breeds at T0, the highest levels were revealed in AV and RE calves in response to the stress caused by the poor welfare of animals at weaning and at the beginning of the fattening phases. In the case of RG calves, they showed the highest haptoglobin values at slaughter time. According to Joshi et al. [58], our results could indicate a higher sensitivity for haptoglobin with respect to SAA, although other authors [21] noted the opposite, indicating that haptoglobin and SAA were unreliable biomarkers of stress for the weaning phase of calves.

In regard to the breeds under study, the AV breed showed the highest haptoglobin value, and for this reason, it could be suggested to be the most sensitive to situations of poor welfare at slaughter time.

The percentage of CRP only affected the RE calves (Figure 4F), reaching the highest value at T50. These results are difficult to explain and could probably be due to individual response factors.

## 4. Conclusions

The results showed that slaughtering was the main cause of acute stress in calves while mixing or non-mixing with unfamiliar animals did not have a significant effect. Breed was identified as the most important factor to be considered in the assessment of stress by serum biomarkers, together with the farm management system (extensive and intensive), and the response assessment of biomarkers was linked to the type of breed. Therefore, the breed and farm management system showed a stronger effect, affecting the majority of biomarkers, on stress situations at slaughter time than pre-slaughter handling (mixing or non-mixing), which only affected the glucose level.

The Retinta breed showed the highest cortisol levels, indicating acute stress throughout the production phase and during the pre-slaughter time, which was probably due to the wilder temperament of this breed. The response of the analyzed serum biomarkers was the opposite of that identified in calves of other breeds, i.e., the Asturiana de los Valles and Rubia Gallega.

The ultimate pH for all the breeds was within the normal range for beef, and this indicator of meat quality was mainly related to stress biomarkers, such as cortisol, lactate, and serum amyloid A. 

Overall, our findings show the complexity of the response to stress in a variety of situations and the multiple blood biomarkers to be considered in their evaluation. Additionally, our results could be useful for the early observation of animals with high levels of stress in order to control their effect on high ultimate pH, and consequently, a greater probability of obtaining DFD meat. Despite the difficulty in selecting a suitable biomarker, our results indicate that cortisol, lactate, and glucose could be reliable stress indicators, but more research is required in order to validate the selected biomarkers as early indicators of stress at slaughtering and their consequences on meat quality.

## Figures and Tables

**Table 1:** Effect of Breed, livestock production system and pre-slaughter handling on serum biomarkers and ultimate pH at slaughtering time (Ts).

	Breed (B)				Farm Management (F)			Pre-Slaughter Handing (PSH)			Interactions			
	AV	RE	RG	*p*-Value	E	I	*p*-Value	M	NM	*p*-Value	B×F	B×PSH	F×PSH	SEM
Cortisol (ng/mL)	251.45 ^b^	341.05 ^a^	230.45 ^b^	0.027	268.54	208.54	0.238	236.62	240.45	0.897	0.004	0.364	0.295	19.648
Lactate (g/L)	0.671 ^b^	1.150 ^a^	0.958 ^a^	0.001	0.919	0.883	0.603	0.937	0.865	0.300	0.001	0.925	0.028	0.0483
Glucose (g/L)	1.112 ^a^	0.895 ^ab^	0.847 ^b^	0.027	1.236	0.666	0.001	1.106	0.901	0.038	0.001	0.148	0.045	0.0659
Serum Amyloid A (µg/mL)	42.69 ^c^	117.20 ^b^	139.21 ^a^	0.001	104.51	106.21	0.828	101.95	108.79	0.382	0.001	0.001	0.046	7.187
Haptoglobin (mg/mL)	0.196	0.164	0.196	0.099	0.326	0.171	0.001	0.221	0.276	0.325	0.001	0.006	0.076	0.0107
CRP test (% positive)	66.67	59.09	75.00	0.469	50.96	62.23	0.232	58.00	54.04	0.662	0.792	0.329	0.606	5.700
pH_u_	5.48 ^c^	5.79 ^a^	5.62 ^b^	0.000	5.63	5.65	0.678	5.64	5.64	0.512	0.512	0.276	0.698	0.017

Values with the same letters (a, b, c) indicate homogeneous subsets for *p* = 0.05 according to Tukey’s test; SEM: Standard Error of Mean; B×F, B×PSH and F×PSH interactions resulted *p* ≥ 0.05 for all parameters. AV: Asturiana de los Valles breed (*n* = 24); RE: Retinta breed (*n* = 22); RG: Rubia Gallega breed (*n* = 32); E: Extensive production system (*n* = 39); I: Intensive production system (*n* = 39); M: Mixing of animals (*n* = 40); NM: No- mixing of animals (*n* = 38).

**Table 2:** Correlation coefficients among serum biomarkers at slaughter time and ultimate pH (pH_u_).

	SAA	Cortisol	Lactate	Glucose	Haptoglobin	pH_u_
SAA	1					
Cortisol	0.12	1				
Lactate	0.342 **	0.430 ***	1			
Glucose	−0.383 ***	0.01	0,04	1		
Haptoglobin	−0.06	−0.02	0.06	0.333 **	1	
pH_u_	0.468 ***	0.400 ***	0.492 ***	−0.15	−0.17	1

SAA: serum amyloid A; ** *p* ≤ 0.01. *** *p* ≤ 0.001.

## Data Availability

None of the data were deposited in an official repository.
